# Supplementary material for: The power of geohistorical boundaries for modeling the genetic background of human populations: The case of the rural catalan Pyrenees
Source: Front Genet. 2023 Jan 10;13:1100440. doi: 10.3389/fgene.2022.1100440 (PMC9871830; doi:10.3389/fgene.2022.1100440)
Supplement: Supplementary file 3 [file DataSheet1.docx]

**The power of geohistorical boundaries for modeling the genetic background of human populations: the case of the rural catalan Pyrenees**

Joan Fibla, Iago Maceda, Marina Laplana, Montserrat Guerrero, Miguel Martín Álvarez, Jesús Burgueño, Agustí Camps, Jordi Fàbrega, Josefina Felisart, Joan Grané, José Luis Remón, Jordi Serra, Pedro Moral and Oscar Lao.

**Supplementary FIGURE. 1-5**


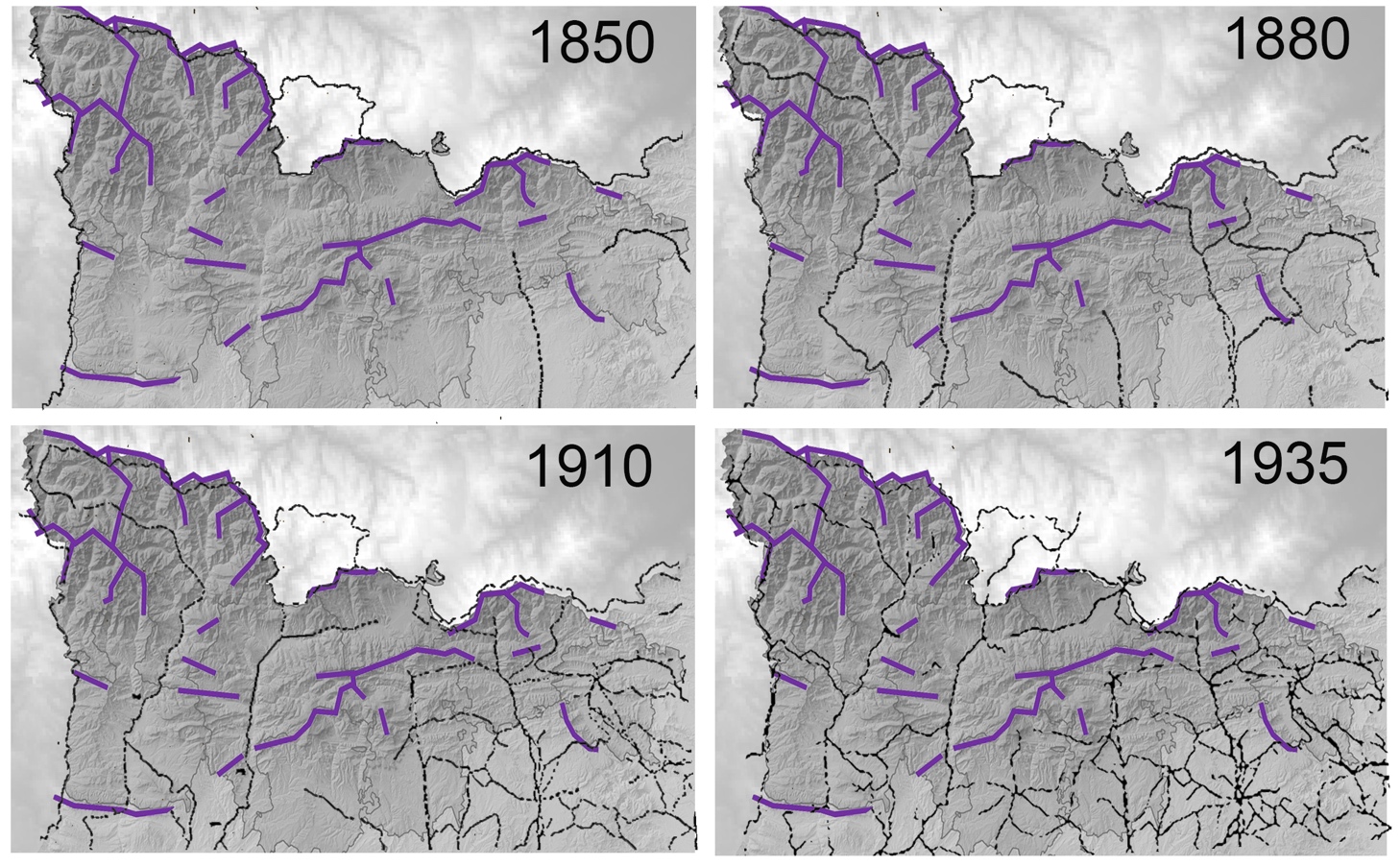


**Supplementary FIGURE. 1.** Evolution of road communications in the catalan Pyrenees over the last two centuries. Black dotted lines designate the growing road network from 1850 to 1935. Purple lines designate the main orographic barriers defined by mountains higher than 1500 meters. Modified from Font 1993.


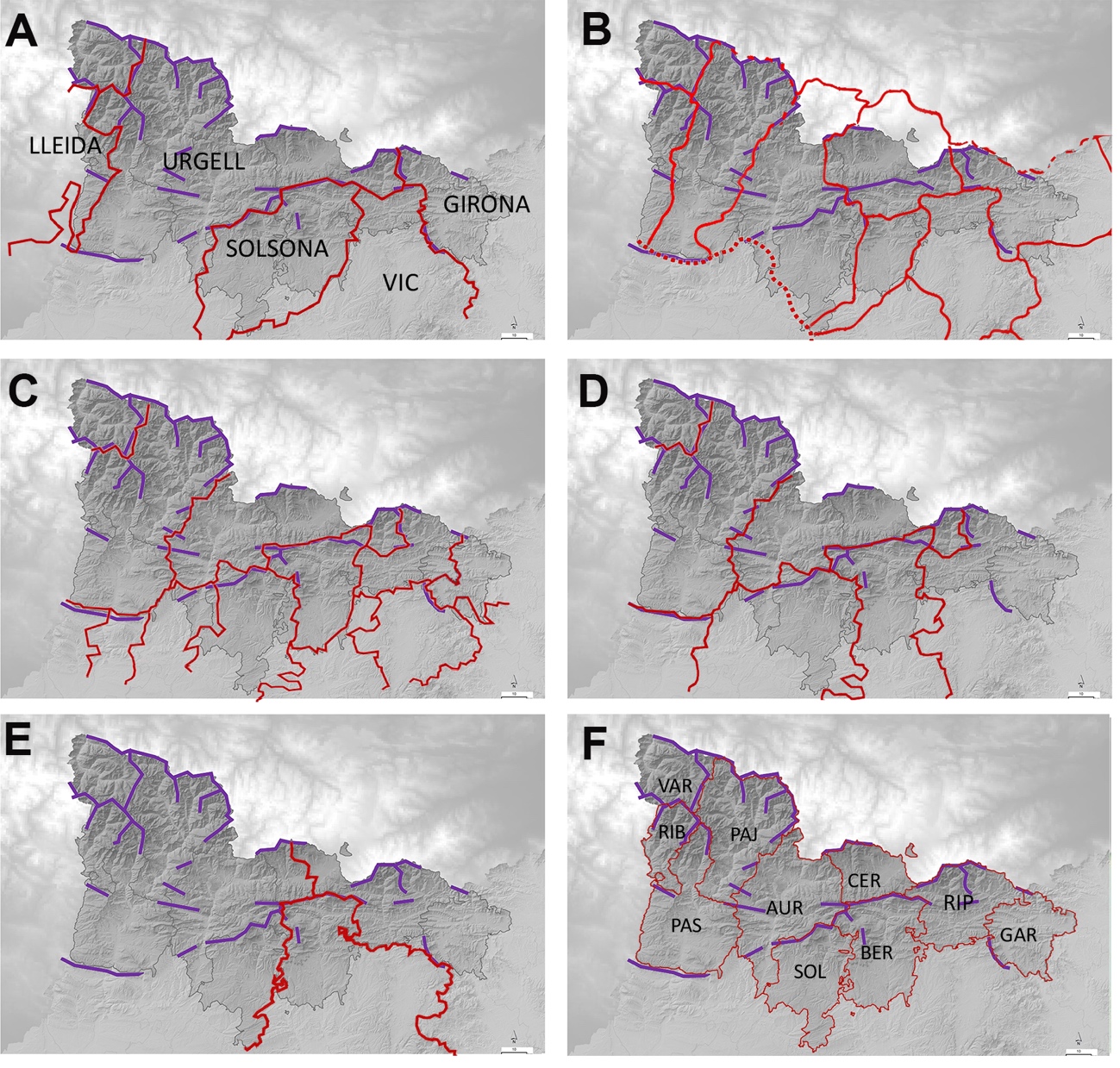


**Supplementary FIGURE. 2.** Historical administrative divisions at the catalan Pyrenees over last centuries. **(A)** Bishoprics boundaries dated on 531 AD to s.XVI. **(B)** Middle-Age County boundaries. **(C)** *Vegueries* boundaries at 12^th^ to 18^th^ centuries. **(D)** *Corregimientos* at 18^th^ century. **(E)** Provinces from 19^th^ century. **(F)** Actual “*comarques*” and Val d’Aran. AUR, Alt Urgell; BER, Berguedà; CER, Cerdanya; GAR, Garrotxa; PAJ Pallars Jussà; PAS; Pallars Sobirà; RIB; Alta Ribagorça; SOL; Solsona; VAR Val d’Aran. Red lines designate administrative-religious borders. Purple lines designate the main orographic barriers. Adapted from Burgeño 1991, 1992.


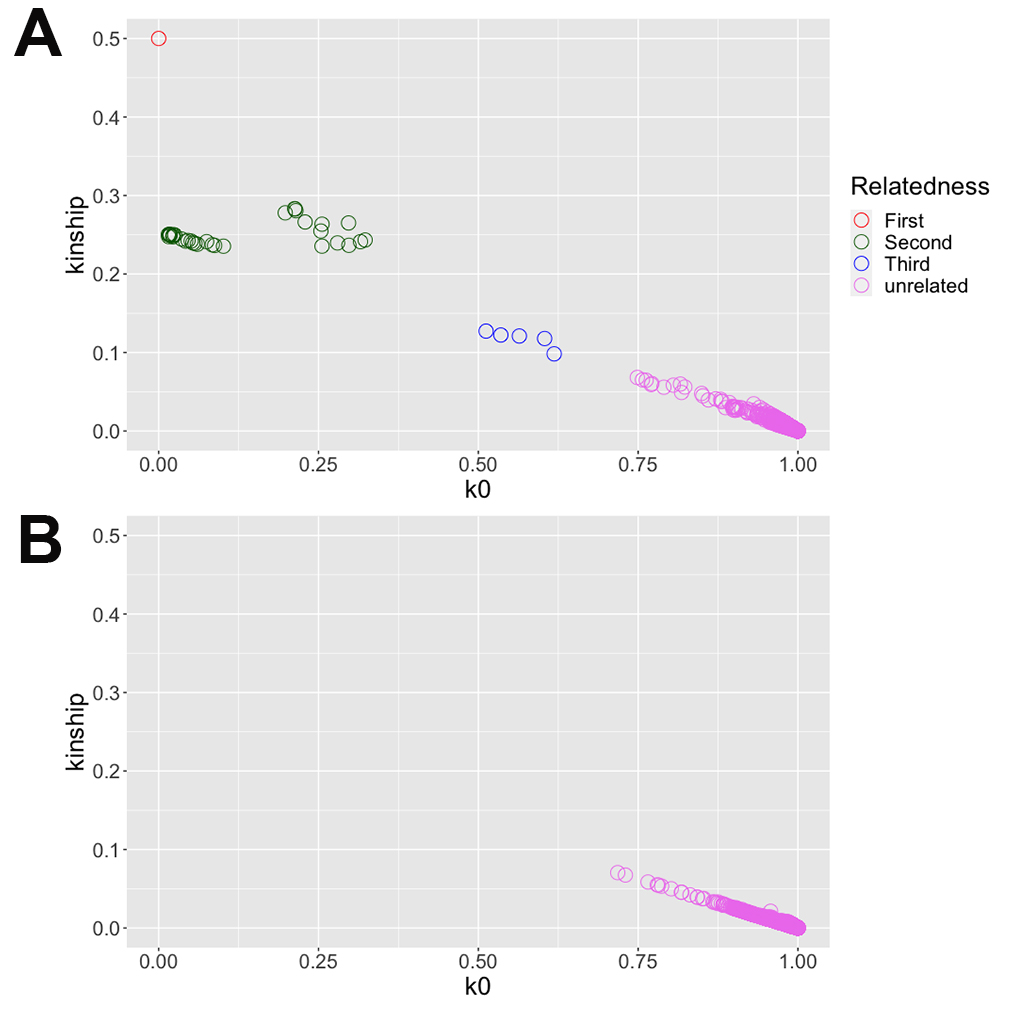


**Supplementary FIGURE. 3**. Relatedness among individual participants. IBD analysis showing k0 vs. kinship values distribution in all 435 genotyped individuals **(A)**, and after excluding those with kinship ≥ 0.09 **(B)**. First degree relatives, (kinship 0.5; k0=0, red circles); second degree relatives (kinship~0.25; k0~0.25, green circles), third degree relatives (kinship~0.25 k0~0.5; blue circles) and unrelated individuals (kinship≤0.09; k0>0.7; pink circles).


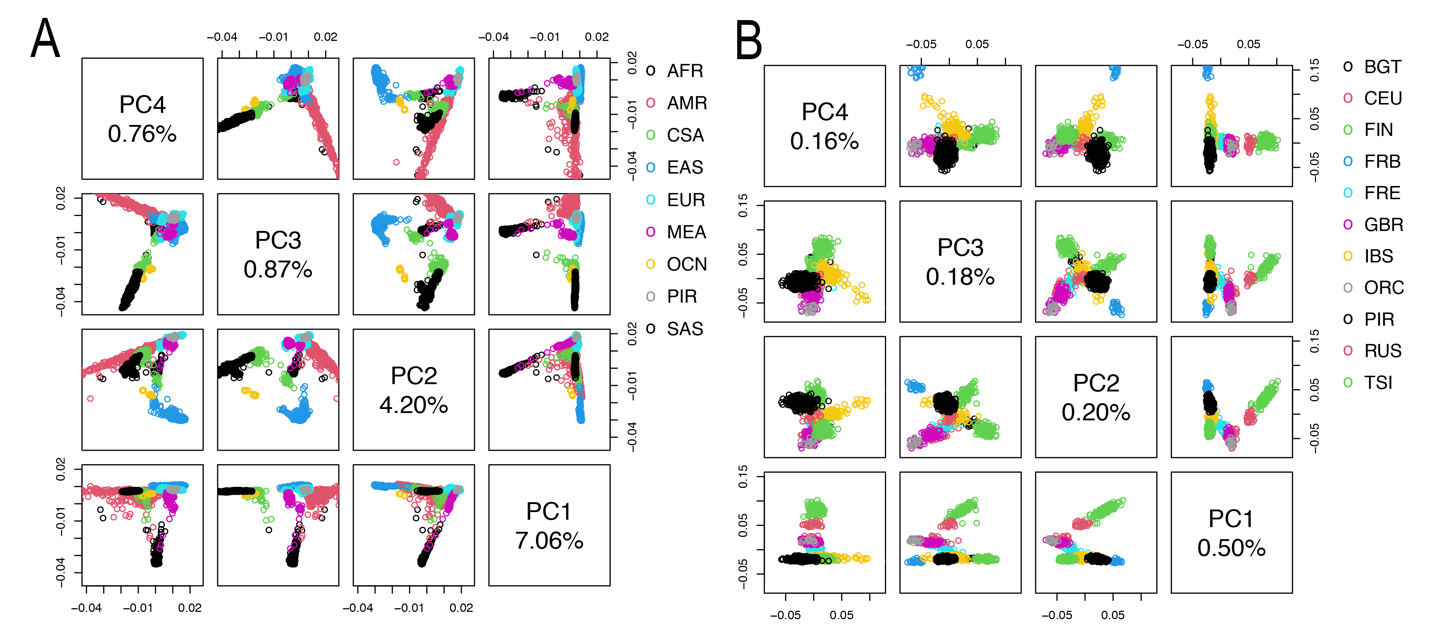


**Supplementary FIGURE. 4**. Scatterplots of the top 4 PCs with proportional variance explained included. (**A**) Top 4 PCs of Merged GENPIR samples and Worldwide samples. (**B**) Top 4 PCs of Merged GENPIR samples and European samples.

**Supplementary FIGURE. 5**. Cross-validation errors (CV) of ADMIXTURE analysis for K=1 to K=4.
